# Comparative Transcriptomic Analysis Provides Insight into Spatiotemporal Expression Patterns of Pivotal Genes During Critical Growth Stages in Min Pig Breed

**DOI:** 10.3390/biom15020180

**Published:** 2025-01-26

**Authors:** Miao Yu, Guandong Wu, Yang Chang, Jiancheng Cai, Chunan Wang, Dongjie Zhang, Chunzhu Xu

**Affiliations:** 1 Key Laboratory of Animal Cellular and Genetics Engineering of Heilongjiang Province, Engineering Research Center of Intelligent Breeding and Farming of Pig in Northern Cold Region, College of Life Science, Northeast Agricultural University, Harbin 150030, China; s230901001@neau.edu.cn (M.Y.); s230902039@neau.edu.cn (G.W.); changy051@neau.edu.cn (Y.C.); 2 China Lanxi Breeding Farm, Lanxi 151500, China; senxu2019@gmail.com (J.C.); z10880@neau.edu.cn (C.W.); 3 Institute of Animal Husbandry, Heilongjiang Academy of Agricultural Sciences, Harbin 150086, China

**Keywords:** Min pigs, growth and development stages, comparative transcriptomics, selection pressure analysis, *CDK2*

## Abstract

The growth and development of animals are dynamic processes characterized by fluctuations. Min pigs, a local breed renowned for their superior meat quality, present an intriguing yet poorly understood relationship between this quality and their growth and development patterns. To elucidate this relationship, we employed a multi-faceted approach that included comparative transcriptomics, quantitative real-time PCR (qRT-PCR), selection pressure analysis of key genes, and three-dimensional protein structure simulations. Our findings revealed that 150 days (150 d) of age represented a pivotal turning point in the growth and development of Min pigs. Thirteen key genes exhibiting significant differential expression between early and late growth stages were identified. Notably, the *CDK2* gene demonstrated specific high expression in the hind limb muscles and adipose tissues during the later growth stages. Comparative analysis with the African warthog revealed that while the CDK2 protein structure remained conserved, base mutations in upstream and downstream non-coding regions resulted in strong positive selection pressure on the *CDK2* gene. These results suggest that *CDK2* plays a crucial role in defining the spatiotemporal characteristics of meat development during the domestication of Min pigs. This study provides critical insights into the growth and development patterns of domestic pigs and offers a robust scientific foundation for improving meat quality traits through domestication.

## 1. Introduction

The growth and development of animals is a complex process that encompasses multiple stages. Studies have shown that many animals, such as insects, exhibit significant phenotypic differences across various growth and development stages. Zhao et al. [1] utilized RNA sequencing (RNA-seq) to identify key genes that play an important role in the growth and molting processes of insects. However, in the growth and development of domestic pigs, where phenotypic differences are not as pronounced, the key functional genes at different stages and their impact on traits remain unknown. Shen et al. [2] determined the growth inflection point (IP) for Liangshan pigs through growth curve analysis, revealing that genes highly expressed during the IP period are primarily involved in energy metabolism and transcriptional activity, which are crucial for rapid growth. Nevertheless, it remains unclear whether an “IP” exists in the growth process of Min pigs and the functions of key genes at different periods. To effectively protect and utilize the high-quality resources of Min pigs, it is necessary to comprehensively investigate the functions of key genes and molecular regulatory mechanisms throughout their developmental stages [3], providing a scientific basis for understanding the growth and development patterns of domestic pigs and improving the genetic characteristics of domestication.

Min pigs are one of the most representative Asian local pig breeds in northern China, known for their excellent meat quality [4]. With rising demand for pork quality, research into the molecular mechanisms that contribute to the superior meat quality traits of Min pigs is intensifying. However, the relationship between the formation of Min pigs’ superior meat quality and their growth and development is still unclear. Studies have demonstrated that genes closely related to the muscle development in livestock and poultry, such as *MYF5*, have time-specific roles in regulating meat quality [5]. Additionally, comparative transcriptome analysis of different beef cutting positions has revealed region-specific expression of functional genes primarily involved in muscle fiber structure, energy production, and conversion processes. These findings indicate that key genes involved in beef quality have spatially specific regulatory roles [6]. However, the key genes associated with the formation of superior meat quality traits in Min pigs, which exhibit spatiotemporal expression characteristics, and their molecular regulatory mechanisms remain unclear. With the advancement of high-throughput sequencing technology, RNA-seq has become a crucial tool for exploring the transcriptional regulatory mechanisms behind key meat quality traits in domestic animals, such as cattle [7,8,9,10], pigs [11,12,13,14], chickens [15,16,17,18], and sheep [19,20,21,22]. Among these techniques, constructing weighted gene co-expression network analysis (WGCNA) using differentially expressed genes (DEGs) obtained from RNA-seq efficiently identifies candidate genes closely associated with specific animal traits [23,24,25,26]. For instance, Wang et al. [27] employed WGCNA to pinpoint potential candidate genes that regulate intramuscular fat content in commercial pigs. Zhang et al. [28] identified key genes and miRNAs that regulate adipocyte differentiation in pigs through WGCNA and K-MEANS analysis. Similarly, Xing et al. [29] used the WGCNA method to identify four modules significantly associated with the backfat thickness in domestic pigs and pinpointed 16 key genes involved in fat deposition. These studies have established a crucial foundation for enhancing meat quality traits in domestic pigs, thereby improving economic benefits. To better understand the relationship between candidate genes and specific traits and enhance genetic improvement, it is essential to explore the selection patterns driven by artificial or environmental factors during animal domestication. Selection pressure analysis at the species level can identify genes influenced by natural selection during domestication. Therefore, integrating WGCNA-selected candidate genes with selection pressure analysis offers a more targeted approach for genetic improvement and optimization of breeding strategies.

In this study, we focused on Min pigs, collecting muscle tissue samples from 36 animals across 12 different growth stages. Using transcriptome sequencing technology, we identified differentially expressed genes and pinpointed the critical transition periods in the growth and development of Min pigs. We applied WGCNA, protein–protein interaction (PPI) network analysis, time series analysis, and key gene selection pressure analysis to explore the molecular genetic basis and gene expression characteristics at various growth stages. These methods facilitated the identification of key functional genes in the growth and development of Min pigs and allowed for the investigation of their biological functions and genetic evolution patterns. This research provides a foundational basis and a candidate gene pool for the enhanced development and utilization of the high-quality genetic resources of Min pigs, as well as for the scientific improvement of growth and meat quality traits in domestic pigs.

## 2. Materials and Methods

### 2.1. Experimental Animals and Sample Collection

The animal samples used in this study were all from the Min pig national breeding farm in Lanxi County, Suihua City, Heilongjiang Province (126°13′17.312″ E, 46°21′48.169″ N). Sampling was conducted from December 2021 to June 2022. Under the standardized breeding conditions at the conservation farm, the project team randomly selected Min pigs from the non-core conservation group in 7 batches across 12 age nodes, namely 30 d, 60 d, 90 d, 120 d, 150 d, 180 d, 210 d, 240 d, 270 d, 300 d, 330 d, and 360 d. At each age node, one boar and two gilts were selected (totaling three biological replicates per node). Following euthanasia, the pigs were immediately slaughtered, and dorsal muscle tissue (DM) was collected. The tissue was rapidly placed in cryovials and submerged in liquid nitrogen and then transported back to the laboratory for storage at −80 °C in a freezer. In addition, during the collection of DM from three 180 d Min pigs, 5 tissues and organs, including the psoas major muscle (PM), subcutaneous shoulder fat (SSF), subcutaneous waist fat (SWF), spleen (SP), and ovary (OV), were collected, which were stored in the same way as described above. After collecting samples from all 12 time points, RNA extraction, detection, qRT-PCR, and transcriptome sequencing were uniformly performed. The work was carried out according to the Regulations for the Administration of Experimental Animals of the People’s Republic of China and was approved by the Ethics and Experimental Animal Committee of Northeast Agricultural University, China (certification No. NEAUEC200104, 31 March 2020).

### 2.2. RNA Extraction, Transcriptome Sequencing, Quality Control, and Quantification of Gene Expression

Total RNA was extracted from 36 muscle tissue samples from Min pigs at 12 different growth stages using TRIzol reagent (Invitrogen, Waltham, MA, USA), followed by quality control. The concentration of RNA samples were measured using an Agilent 5400 analyzer (Agilent, Santa Clara, CA, USA), and agarose gel electrophoresis was employed to check for DNA contamination. Subsequently, they were sent to Shanghai Majorbio Bio-Pharm Technology Co., Ltd. for re-assessment and subsequent transcriptome sequencing. RNA-seq libraries were constructed and sequenced on the Illumina HiSeq sequencing platform using second-generation sequencing technology for paired-end sequencing. Each sample was aligned to the pig reference genome (*Sus scrofa* 11.1) to complete genomic localization analysis. The number of uniquely mapped reads for each specific transcript was counted using HTSeq (v0.6.1) [30]. The number of reads matching was normalized, and the standardization method used was fragments per kilobase per million reads fragments (FPKM) [31]. It is generally considered that in eukaryotic reference transcriptomes, transcripts with FPKM values greater than 1 correspond to expressed genes.

### 2.3. Differential Expression Genes Detection

We conducted differential expression analysis on gene expression data from Min pigs across 12 different growth stages, utilizing the DEGSeq tool on the Majorbio Cloud Platform (https://cloud.majorbio.com/, accessed on 23 April 2024) [32] to identify DEGs. The selection criteria were |log2Fold Change| > 1 and Q-value < 0.05, where the Q-value is the *p*-value adjusted by the Benjamini–Hochberg (BH) method, which better controls the false positive rate. We used the Cluster Heatmap tool on the Microbioinformatics Online website (https://www.bioinformatics.com.cn, accessed on 23 April 2024) for hierarchical clustering analysis of DEGs from Min pigs at ages 30 d to 360 d, employing the complete linkage algorithm and Euclidean distance method.

### 2.4. Weighted Gene Co-Expression Network Analysis and Core Module Identification

WGCNA [33] partitions genes into highly correlated modules based on their expression patterns, using the module eigengene (ME) to represent the expression characteristics of each module. This approach allows for the assessment of module membership and ultimately evaluates the correlation between these modules and the phenotypic traits of interest. In this study, we used the FPKM values of gene expression derived from standardized transcriptome sequencing data of Min pigs at various stages. The normalization of all sample transcriptome data was performed using the WGCNA shiny plugin based on the R package in TBtools (v1.09) [34]. In the construction of the co-expression network, genes with FPKM values greater than 1 were selected, totaling 10,550 genes. Before network construction, potential outliers in the samples were excluded. By setting the R2 threshold to 0.8, the soft threshold (Power) that first reached the 0.8 numerical line was selected, and the appropriate Power value of 7 was determined based on scale independence. In the modular analysis, a merge cutting threshold of 0.25 was set, and the minimum module size was stipulated to include at least 50 genes. The clustering tree was divided into different gene modules using the dynamic branch cutting method. Each module represents a set of genes with similar expression patterns, which are indicated by different colors in the diagram. The module eigengene (ME) is defined as the first principal component of the module of interest and can be considered as a representative of the module’s gene expression profile. We screened modules significantly related to the growth stages of Min pigs with a criterion of correlation coefficient *r* > 0.8 and *p* < 0.01, where the module eigengenes are considered important gene sets.

### 2.5. Core Module Genes Pathway Enrichment Analysis

Leveraging the Kyoto Encyclopedia of Genes and Genomes (KEGG) database [35], we analyzed metabolic processes and pathway networks within biological systems. Pathway significance enrichment analysis helped us identify the main biochemical metabolic processes and signal transduction pathways involving core module genes. Using the DAVID database platform (https://davidbioinformatics.nih.gov/, accessed on 14 May 2024), we conducted enrichment analyses based on the KEGG pathways corresponding to our genes of interest. We sorted the enrichment results by significance in descending order with a Q-value threshold of <0.05. For the top 20 significant KEGG pathways, visualization was performed using the Enrichment Dot Bubble tool on the Microbioinformatics Online platform.

### 2.6. Core Genes Selection

To identify genes most associated with the growth and development of Min pigs during 2 critical stages, (1) we considered each period as a whole, obtained non-differentially expressed genes (|log2Fold Change| < 1) for the comparisons of 30 vs. 60 d, 60 vs. 90 d, and 90 vs. 120 d and defined the intersection of these genes as the non-differentially expressed gene set for the early growth and development stage of Min pigs; Similarly, we obtained non-differentially expressed genes for the comparisons of 150 vs. 180 d, 180 vs. 210 d, 210 vs. 240 d, 240 vs. 270 d, 270 vs. 300 d, 300 vs. 330 d, and 330 vs. 360 d and defined the intersection of these genes as the non-differentially expressed gene set for the later growth and development stage of Min pigs. Subsequently, the intersection genes of non-differentially expressed genes from the early and late stages were obtained. (2) We performed differential expression analysis on the intersection genes using the DEGSeq tool from the Majorbio Cloud Platform, with the selection criteria of |log2Fold Change| > 1 and Q-value < 0.05, to obtain the DEG dataset. (3) The intersection genes of the core module genes from both stages and the DEG dataset were considered as the candidate gene dataset most related to the two growth stages of Min pigs. (4) Gene Ontology (GO) is applicable to various species, primarily functions to annotate and describe the functions of genes and proteins [36]. We used the DAVID database platform to perform GO and KEGG enrichment analysis on the candidate gene set and raised the selection threshold for GO and KEGG to identify the most significant GO terms and KEGG pathways with *p* < 0.01, obtaining genes within GO terms and KEGG pathways with *p* < 0.01. (5) By taking the intersection of genes within GO terms and KEGG pathways with *p* < 0.01, we obtained the core gene set most related to the two growth stages of Min pigs.

### 2.7. Core Genes Time-Series Analysis

To better understand the regulatory role of core genes in the growth and development of pigs, a time-series analysis of these core genes at 12 consecutive growth nodes from 30 d to 360 d was conducted. Mfuzz [37] is capable of identifying potential time series patterns in expression profiles and clustering genes or proteins with similar patterns, thereby understanding the dynamic change patterns and functional connections of genes or proteins. Based on the gene expression data of core genes at 12 nodes in transcriptome sequencing data, the Mfuzz TimeSeries tool from the online bioinformatics website was used to visualize the expression data of core genes at various periods using the fuzzy C-means clustering algorithm.

### 2.8. Protein-Protein Interaction Network Analysis

The Search Tool for the Retrieval of Interacting Genes (STRING) [38] (https://string-db.org/, accessed on 22 May 2024) database is primarily used for analyzing protein–protein interactions and providing information on protein domains and three-dimensional structures. As a tool for filtering and assessing functional genomics data, it provides an integrated network information platform for the structural and functional annotation of proteins. Core gene interaction data is extracted from the STRING database and exported in TSV format for visualization in Cytoscape (v3.9.1) [39] software. Cytoscape is a tool for integrating molecular interaction networks, gene expression data, and molecular biology information. Its core functions include basic network layout, large-scale protein–protein interaction analysis, network querying, and network graph beautification. The network graph includes nodes formed by genes and proteins, connections between nodes (edges), and the important topological property—degree, which is defined as the number of connections to a node. The degree values of core network genes are calculated using the cytoHubba plugin in Cytoscape to identify hub genes that play a key role in the gene interaction network. To determine the main candidate genes related to growth and development, we set the degree value ≥ 4 for core network genes as important candidate genes.

### 2.9. qRT-PCR

Total RNA was extracted from six tissues and organs of pigs at 180 d of age, including DM, PM, SSF, SWF, SP, and OV. All extracted RNA samples were reverse transcribed using a reverse transcription kit (Vazyme, Nanjing, China) to obtain cDNA. Thirteen identified core genes were selected for qRT-PCR experiments with GADPH as the reference gene. Primers were designed using Primer 5 [40] and synthesized by Genewiz Biotechnology Co., Ltd. (https://www.genewiz.com.cn/, accessed on 29 May 2024) (Table S1). qRT-PCR analysis was conducted using the LightCycler^®^96 system (Roche, Switzerland). The qRT-PCR reaction system consisted of 10 μL, containing 5 μL of SYBR fluorescent dye, 1 μL of cDNA, 3.4 μL of sterile water, and 0.3 μL each of forward and reverse primers. The reaction conditions were as follows: 95 °C for 10 min for initial denaturation, 95 °C for 15 s for denaturation, 60 °C for 60 s for annealing, with 40 cycles. Melting curve analysis indicated that each PCR product was a single peak. The relative expression levels of mRNA were calculated using the $2^{-△△CT}$ method. Each tissue sample was subjected to three biological replicates, with at least three technical replicates for each biological replicate for subsequent expression analysis.

### 2.10. Selection Pressure Analysis

Gene selection pressure can be categorized into three types based on the ω (Ka/Ks) ratio: ω < 1 represents negative selection; ω > 1 represents positive selection; and ω = 1 represents neutral selection. To detect the selection pressure on 13 core genes in pigs, we selected Min pigs, *Phacochoerus africanus*, *Bison bison*, and *Cervus elaphus* for selection pressure analysis. The corresponding gene sequences of *P. africanus*, *B. bison*, and *C. elaphus* were obtained from the NCBI database (https://www.ncbi.nlm.nih.gov/, accessed on 20 June 2024) for use in branch and site model tests. Maximum likelihood (ML) methods were used to construct phylogenetic trees for the 13 candidate genes, and EasyCodeML 1.4 [41] was utilized to calculate the ω values of each Min pigs’ genes relative to the selected artiodactyl animals in the phylogeny. Site models within this software were used to test the hypothesis of positive selection for the candidate genes, allowing selection pressures to vary with different sites, assuming that different branches of the phylogenetic tree experience the same selection pressure, but different amino acid sites experience different selection pressures. Subsequently, likelihood ratio tests (LRTs) were performed, and when LRTs were significant (*p* < 0.05), the Bayes Empirical Bayes (BEB) method was employed to select positively selected sites with posterior probabilities > 99%. The identified sites were further validated and supplemented using the mixed effects model (MEME) and fixed effects model (FEL) in the DataMonkey [42] website (http://www.datamonkey.org/analyses, accessed on 28 July 2024), with sites satisfying β+ > α and likelihood ratio test (LRT) *p* < 0.1 being identified as positively selected sites.

### 2.11. Core Genes Protein Three-Dimensional Structure Simulation

To determine the three-dimensional structure of the *CDK2* gene protein under positive selection, the protein sequence of the *CDK2* gene in *P. africanus* was obtained from the NCBI database. ColabFold (v1.5.3) [43] was utilized to predict the three-dimensional structure of the protein based on the protein sequences of Min pigs and *P. africanus*. Using the Duroc CDS sequence as a reference, sequences upstream and downstream of the Min pigs and *P. africanus CDK2* genes, each 138 bp, were converted into protein sequences and their three-dimensional structures were predicted. It is important to note that the region 130–132 bp upstream is the upstream in-frame stop codon; therefore, the region 1–129 bp upstream was selected for the prediction of the protein’s three-dimensional structure. PyMOL [44] (https://pymol.org, accessed on 10 August 2024) was used to visualize and output the three-dimensional structure of the protein.

## 3. Results

### 3.1. Identification of Key Transitional Periods in the Growth and Development of Min Pigs

**Figure 1:**
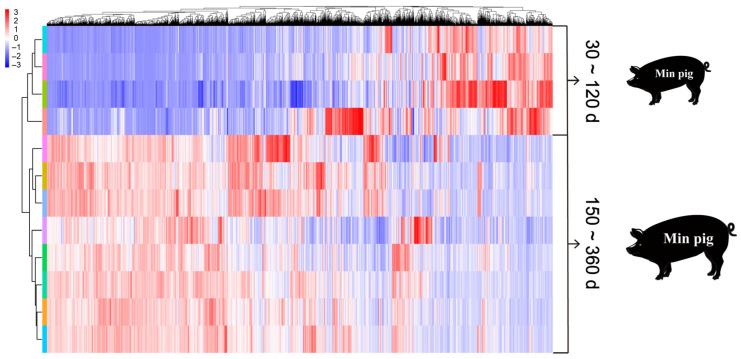
Hierarchical clustering diagram of DEGs in Min pigs from 30 d to 360 d. The deeper the blue, the lower the relative gene expression; the deeper the red, the higher the relative gene expression.

Hierarchical cluster analysis was performed on DEGs in Min pigs from 30 d to 360 d of age (Figure 1). The results indicated that Min pigs from 30 d to 360 d were divided into two distinct developmental stages based on the similarity of gene expression patterns: samples from 30 d to 120 d and from 150 d to 360 d each formed a separate cluster. This also confirmed that 150 d is a critical turning point in the growth and development process of Min pigs. Based on the sequence of development, these stages were defined as the early stage (E stage, 30 d to 120 d) and the late stage (L stage, 150 d to 360 d) of growth in Min pigs.

### 3.2. Selection of Core Genes at Different Stages of Growth and Development in Min Pigs

**Figure 2:**
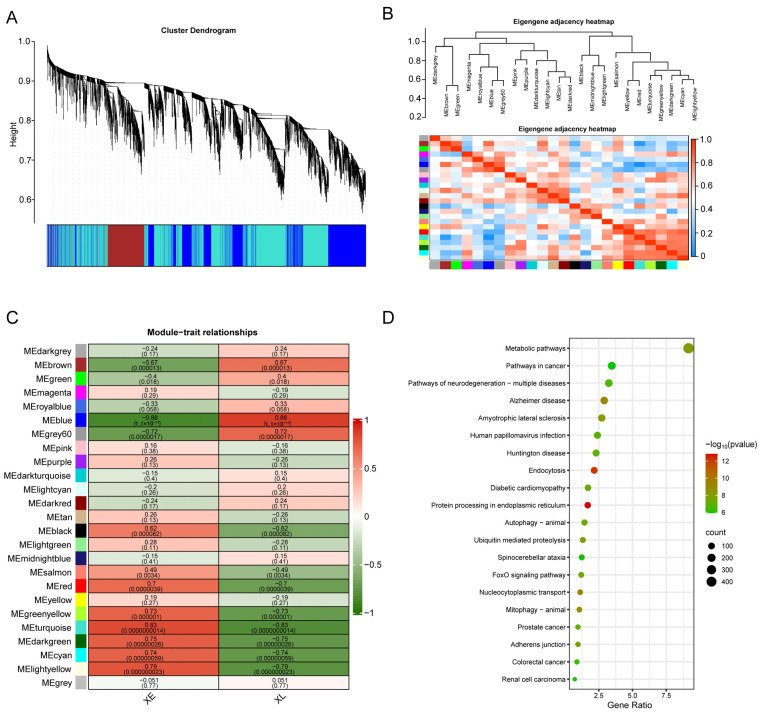
Analysis of gene co-expression modules and their functional enrichment. (**A**) Cluster dendrogram showing each branch as a gene and each color representing a different co-expression module. (**B**) Hierarchical clustering and module correlation heatmap of the modules. Different colors at the bottom represent different modules. (**C**) The relationship between co-expression modules and growth traits (growth stages), where the *x*-axis represents sample information for the two growth stages, and the *y*-axis represents each gene module. In the panel, the deeper the color, the higher the correlation; red indicates positive correlation, and green indicates negative correlation. The first row of data in each cell is the correlation between the module and the stage, and the second row is the significance (*p*-value). (**D**) KEGG functional enrichment of genes in key modules.

Based on the RNA-Seq data from Min pigs, gene expression levels were analyzed using WGCNA. By calculating the co-expression correlation coefficients among genes, a total of 25 co-expression modules were constructed (Figure 2A). The gene expression trends within the 25 modules were clustered, analyzed for correlation, and then correlated with the growth traits (growth stages) of Min pigs. The results showed that among the 25 modules, two modules significantly correlated with the growth stages of Min pigs were identified with a criterion of correlation coefficient *r* > 0.8 and *p* < 0.01. These were the MEturquoise module, which was significantly positively correlated with the early stage (*r* = 0.83, *p* < 0.01), and the MEblue module, which was significantly positively correlated with the late stage (*r* = 0.88, *p* < 0.01) (Figure 2B,C). A total of 5066 genes were enriched in these two modules. To further understand the functions of the genes within these two modules, a KEGG enrichment analysis was performed on all genes contained in the modules. Among the top 20 enriched pathways, significant pathways included protein processing in the endoplasmic reticulum, mitosis, amyotrophic lateral sclerosis, metabolic pathways, ubiquitin-mediated protein degradation, autophagy, and the FoxO signaling pathway, most of which are closely related to organismal growth and metabolism (Figure 2D).

**Figure 3:**
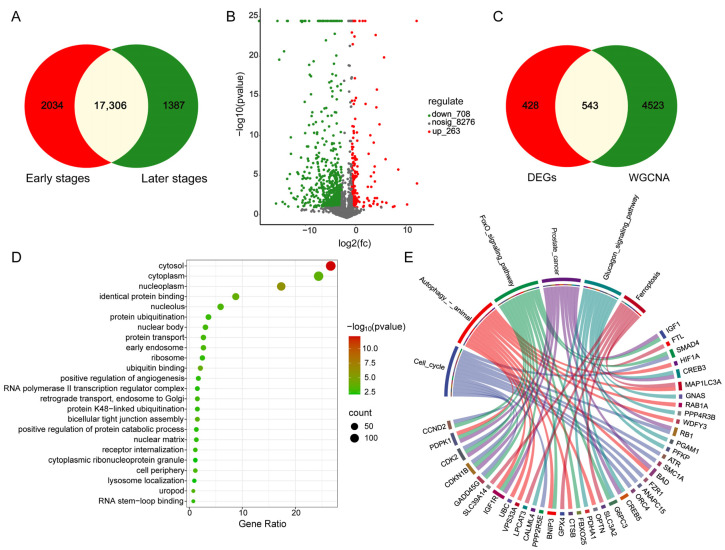
Differential expression analysis of transcriptomes between two growth stages in Min pigs and screening of key functional genes. (**A**) Intersection of non-differentially expressed genes between the early and late stages. (**B**) Volcano plot of gene expression profiles, where red represents upregulated genes, green represents downregulated genes, and gray represents non-differentially expressed genes. The horizontal axis log2 (fc) indicates the fold change of differential expression, and the vertical axis -log10 (*p*-value) indicates the significance of differential expression. (**C**) Intersection of DEGs with WGCNA core module genes. (**D**) GO enrichment analysis of the intersection genes, displaying the top 24 GO terms (*p* < 0.01). (**E**) KEGG enrichment analysis of the intersection genes, displaying the top 6 KEGG pathways (*p* < 0.01) and the genes they contain.

Based on the FPKM values from comparative transcriptome sequencing data, differentially expressed gene analysis was performed, identifying 17,306 shared genes between the early and late stages that were not differentially expressed (Figure 3A). Among these, 263 genes were upregulated, and 708 genes were downregulated (Figure 3B). There were 543 shared genes between the DEGs (971) between the two growth stages and the WGCNA-enriched gene set (5066) (Figure 3C). GO and KEGG enrichment analysis of the shared genes revealed that 24 GO terms were significantly enriched (*p* < 0.01) (Figure 3D), including 335 DEGs; 6 KEGG pathways were significantly enriched (*p* < 0.01), namely the FoxO signaling pathway, cell cycle, prostate cancer, autophagy—animals, ferroptosis, and glucagon signaling pathway (Figure 3E), including 40 DEGs.

### 3.3. Expression Temporal Characteristics of Key Genes in Important Stages of Min Pigs Growth and Development

**Figure 4:**
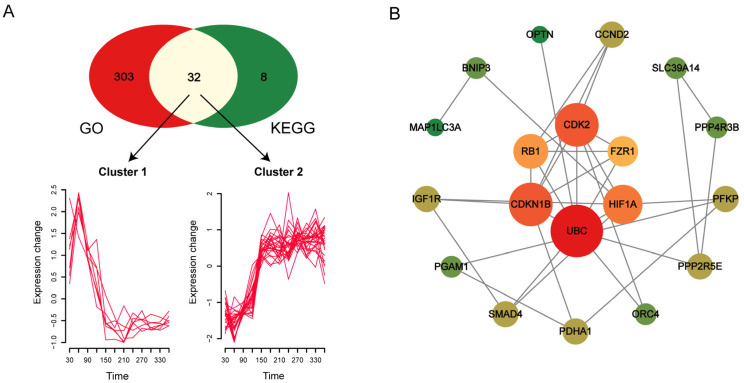
Temporal analysis and protein–protein interaction network analysis of key genes. (**A**) Temporal analysis of core genes. The Venn diagram above shows the intersection of genes from GO and KEGG enrichment analyses with *p* < 0.01 as important candidate genes, and the temporal analysis below shows the divided two clusters of intersecting genes, with the horizontal axis representing different ages and the vertical axis representing changes in relative expression levels. (**B**) Protein–protein interaction network diagram of the second cluster of genes from the temporal analysis, where the deeper the red, the higher the degree value, and the deeper the green, the lower the degree value.

To more accurately identify key functional genes affecting the growth and development of Min pigs, the gene sets with *p* < 0.01 from GO terms were intersected with those in KEGG pathways, resulting in a total of 32 shared genes. A time-course analysis of the expression levels of these 32 genes at different ages showed that they were divided into two clusters (Figure 4A). The first cluster, which included *SLC3A2*, *FTL*, *GPX4*, *MAP1LC3A*, *BAD*, *BNIP3*, and *GADD45G*, exhibited high expression in the early stage and low expression in the late stage. The second cluster, containing 25 genes such as *CDK2*, *CDKN1B*, *RB1*, and others, showed low expression in the early stage and high expression in the late stage. KEGG functional enrichment analysis of the two clusters revealed that the first cluster of genes was mainly enriched in ferroptosis and autophagy pathways, while the second cluster of genes was mainly enriched in cell cycle, autophagy, and FoxO signaling pathways (Figures S1 and S2). Protein–protein interaction network analysis of the 25 candidate genes in the late growth stage showed that 6 core genes, namely *CDK2*, *CDKN1B*, *RB1*, *FZR1*, *UBC*, and *HIF1A*, were identified when the degree value was ≥4 (Figure 4B).

### 3.4. qRT-PCR Validation in Different Tissues and Organs of Min Pigs

**Figure 5:**
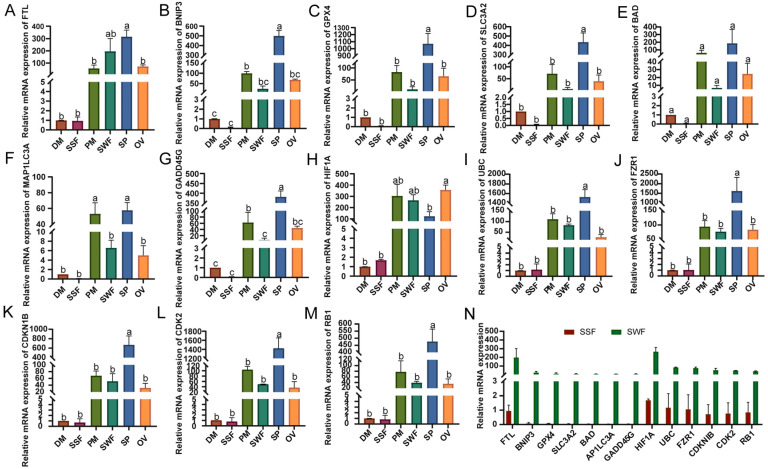
qRT-PCR results of core genes in various tissues and organs of Min pigs, where DW represents the dorsi muscle, SSF represents the subcutaneous shoulder fat, PM represents the psoas major muscle, SWF represents the subcutaneous waist fat, SP represents the spleen, and OV represents the ovary. (**A**–**M**) The qRT-PCR results for different core genes are shown, with the vertical axis representing the relative mRNA expression levels of the genes and the horizontal axis representing the different tissues and organs. (**N**) The expression patterns of different core genes in SSF and SWF, with the vertical axis representing the relative mRNA expression levels of the genes in these two sites and the horizontal axis representing the different genes. The significant difference analysis was conducted using the letter marking method.

The expression levels of the 13 key genes identified in six tissues and organs of Min pigs (SP, SWF, SSF, OV, DM, and PM) are shown in Figure 5A–M. Except for *HIF1A*, all other genes exhibited the highest relative expression levels in the SP. Moreover, the relative expression levels of the 13 genes in the PM and SWF were higher than those in the DW and SSF, indicating a typical spatial (forelimb and hindlimb) differential expression pattern among the 13 key genes in the fat and muscle tissues of Min pigs (Figure 5N). This suggests that lipid deposition in Min pigs is mainly concentrated in the development of the hindlimbs during the late stages of growth and development.

### 3.5. Analysis of Selection Pressure at Key Stages of Growth and Development in Min Pigs

To further elucidate the selection pattern characteristics of the 13 identified genes (*CDK2*, *CDKN1B*, *RB1*, *FZR1*, *UBC*, *HIF1A*, *SLC3A2*, *FTL*, *GPX4*, *MAP1LC3A*, *BAD*, *BNIP3*, *GADD45G*) affecting growth and development in Min pigs, we selected Min pigs, *P. africanus*, *B. bison*, and *C. elaphus* for selection pressure analysis. The results indicated that the *CDK2* gene was under typical positive selection (ω > 1), while the remaining 12 candidate genes were under typical negative selection (ω < 1) (Table 1).

### 3.6. Simulation of the Three-Dimensional Structure of the CDK2 Gene at Key Stages of Growth and Development in Min Pigs

**Figure 6:**
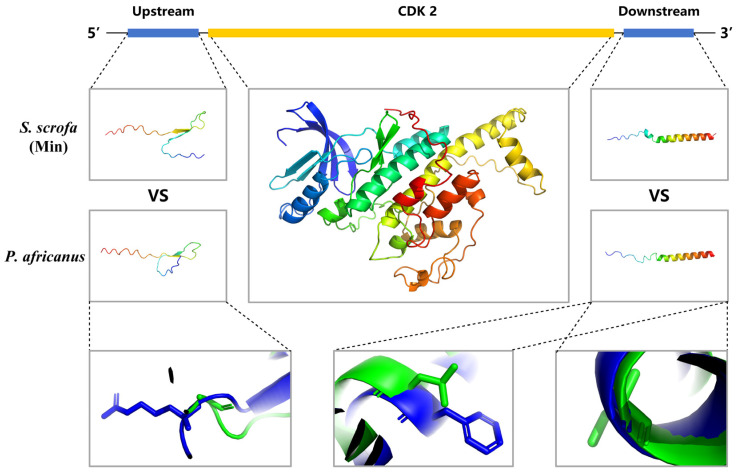
*CDK2* gene structure and three-dimensional protein structure simulation of the *CDK2* gene in Min pigs and *P. africanus*. In the three detailed figures below, the first detail figure illustrates the differences in amino acid structures in the upstream non-coding protein sequences of the *CDK2* gene between Min pigs and *P. africanus*, while the second and third detail figures show the differences in amino acid structures in the downstream non-coding protein sequences of the *CDK2* gene between Min pigs and *P. africanus*, with blue representing Min pigs and green representing *P. africanus*.

By comparing the three-dimensional structures of the proteins encoded by the *CDK2* gene between Min pigs and *P. africanus*, it was found that the protein structures were identical. However, significant differences were observed in the three-dimensional structures of the upstream and downstream non-coding sequences (Figure 6). Specifically, a base mutation upstream (from G to C) resulted in a change from Gly (in *P. africanus*) to Arg (in Min pigs); two base mutations downstream (from C and A to T and G) led to a change from Leu and Ser (in *P. africanus*) to Phe and Gly (in Min pigs). This phenomenon is considered to reflect the selection of regulatory factors during the domestication process of pigs, suggesting that the *CDK2* gene has undergone selection.

## 4. Discussion

In the field of pig breeding, meat quality is a crucial economic trait. This study utilized WGCNA to identify candidate genes associated with different developmental stages in Min pigs and analyzed the selection patterns and intensities of these candidate genes in various wild artiodactyl populations. It was found that the *CDK2* gene plays an important role during the late growth and development stages of Min pigs. Sun et al. identified *CDK2* as a critical protein kinase essential for regulating lipid deposition, acting through srebf1, a key transcription factor for adipogenesis, as shown by network analysis [45]. Furthermore, Jiang et al. discovered that interfering with FOXO1 promoted the proliferation of bovine skeletal muscle cells (BSMC) by upregulating the expression of *CDK2* [46]. These studies indicate that *CDK2* is regulated by multiple factors, which affect body fat deposition and muscle development in animals. In this study, we found that the *CDK2* gene was significantly upregulated during the late growth stages in Min pigs, exhibiting time-specific high expression characteristics. This suggests that *CDK2* may play an important role in the development of muscle and adipose tissues at this stage. In addition, past studies have shown that genes, such as *RB1* [47], *UBC* [48], *HIF1A* [49], *CDKN1B* [50], and *FZR1* [51], also participate in the regulation of lipid metabolism, either directly or indirectly. Additionally, these genes are significantly upregulated during the later growth stages in Min pigs. Therefore, lipid deposition in the growth and development of Min pigs may primarily occur in the later stages.

It is noteworthy that the expression of the *CDK2* gene is higher in the hindlimb muscles and adipose tissues than in the forelimb, exhibiting spatially specific high expression characteristics. Similarly, several other key genes also show similar expression patterns. From an evolutionary perspective, the *P. africanus*, the most widely distributed species of wild boar [52], has developed forelimbs more robust than its hindlimbs due to its long-term survival in complex environments, aiding in hunting prey. In contrast, domestic pigs, subjected to prolonged artificial selection, show significantly greater muscle and adipose tissue content in their hindlimbs than in their forelimbs [53], which aligns with the demands for higher pork quality. Selection pressure analysis is an effective tool for understanding gene evolutionary processes, providing insights into how genes respond to natural selection and environmental changes, thereby revealing their roles and significance in the evolutionary process [54]. In this study, selective pressure analysis was conducted during the domestication process of Min pigs and several representative species in the order Artiodactyla at the species level. The *CDK2* gene was found to be under positive selection, highlighting its importance in the genetic evolution of Min pigs. This also indicates that *CDK2* is closely related to the artificial domestication process of pigs. A comparison of the three-dimensional protein structures of the *CDK2* gene between Min pigs and *P. africanus* revealed no differences, suggesting that the positive selection effect on this gene during domestication was not due to changes in the protein’s structure itself. However, differences were found in the three-dimensional structures of the upstream and downstream non-coding protein sequences between Min pigs and *P. africanus*.

Studies have shown that the 5′ untranslated region (5′-UTR) of the mRNA upstream of the coding sequence (CDS) may contain open reading frames (uORFs) with translational capabilities, which can inhibit the translation of main open reading frames (mORFs) through various mechanisms [55]. In this study, codon changes within uORFs led to the replacement of Gly with Arg during domestication. Gly lacks a side chain, which contributes to protein flexibility [56], while Arg has a long positively charged side chain that may affect protein charge distribution and interactions with other molecules [57]. From an evolutionary perspective, such changes may have significant impacts on protein function and are thus preserved throughout evolution. Researchers have found that the evolution of uORFs is driven by positive selection, suggesting that codon changes may confer some adaptive advantage [55]. Additionally, the use of common codons can enhance mRNA stability and translation efficiency [58]. Additionally, as elements of translational regulation [59], codon changes may help fine-tune gene expression to adapt to different environmental conditions. Studies have shown that sequences around the initiation codon affect translation initiation and early elongation [60]; therefore, codon changes in the non-coding protein sequence near the upstream promoter in Min pigs may affect protein translation, subjecting the *CDK2* gene to positive selection during domestication. Furthermore, the 3′ untranslated region (3′-UTR) downstream of the CDS contains many cis-regulatory elements, such as AU-rich elements and RNA-binding protein binding sites, which may bind to trans-acting factors to regulate mRNA stability and translation processes [61]. Studies have indicated that downstream open reading frames (dORFs) may affect mRNA stability [62], and their translation products may be involved in post-translational regulation of proteins [63], such as forming protein complexes or affecting the stability and activity of other proteins [64]. In this study, base mutations in the 3′-UTR region may affect the translation of dORFs and thus influence post-translational regulation, subjecting the *CDK2* gene to positive selection during domestication. However, to accurately understand the impact of these changes, further research is required to determine their effects.

In summary, the *CDK2* gene may play a key role in the quality traits of meat during the later stages of growth and development in Min pigs by regulating lipid deposition and muscle development, and it may be a key gene in shaping the spatiotemporal characteristics of meat development. This finding provides a foundational basis and a candidate gene pool for better development and utilization of the high-quality genetic resources of Min pigs, as well as for the further scientific improvement of growth traits and meat quality in domestic pigs. The next step of this study will delve into the specific molecular mechanisms by which the *CDK2* gene regulates lipid metabolism in domestic pigs, providing new molecular marker tools for the precision genetic improvement of domestic pigs.

## 5. Conclusions

Transcriptome sequencing data revealed that 150 d of age is a critical turning point in the growth and development of Min pigs, with 13 key genes identified that show significant differential expression between the early and late stages of growth and development. Among these, the *CDK2* gene shows specific high expression in the hindlimb muscles and adipose tissues during the later stages of growth and development. Compared with *P. africanus*, although the CDK2 protein structure is the same, base mutations in the upstream and downstream non-coding protein sequences result in a strong positive selection effect on the *CDK2* gene. Therefore, it is reasonable to believe that the *CDK2* gene is a key gene in shaping the spatiotemporal characteristics of meat development during the domestication process of Min pigs. This study provides feasible strategies for making full use of the excellent meat quality traits of Min pigs to enhance economic benefits.

## Figures and Tables

**Table 1:** Selection pressure analysis of 13 candidate genes.

Gene	−2∆lnL	PAML	MEME (*p* < 0.1)	FEL (*p* < 0.1)	All Sites	ω
*CDK2*	0.03380	—	—	—	0	1.38998
*CDKN1B*	6.80000	—	—	—	0	0.65479
*RB1*	35.07018	455, 459, 462, 467, 469, 471, 475, 480, 481, 483, 493, 495, 496, 498, 500, 502, 507, 508, 509, 511, 513, 515, 518, 520, 527, 534, 543, 544, 552, 554, 556, 558, 562, 567	—	455, 456, 490, 516, 517, 524, 529, 540, 553, 584	43	0.48101
*FZR1*	0.00222	—	—	—	0	0.01758
*UBC*	13.04188	—	—	—	0	0.08146
*HIF1A*	1.41780	—	355	—	1	0.00010
*SLC3A2*	7.64715	343	5, 7, 10, 19, 23, 24, 42, 273	3, 5, 7, 8, 10, 19, 21, 23, 39, 41, 42, 45	15	0.31304
*FTL*	1.50000	—	—	—	0	0.47354
*GPX4*	8.03132	—	93	—	1	0.72971
*MAP1LC3A*	6.41036	—	—	—	0	0.12543
*BAD*	0.03784	—	28, 152	28, 152	2	0.67307
*BNIP3*	3.86000	—	—	69	1	0.91521
*GADD45G*	0.04207	—	6	—	1	0.78297

## Data Availability

The data presented in this study are available on request from the corresponding author.

## Supplementary Materials

The following supporting information can be downloaded at: https://www.mdpi.com/article/10.3390/biom15020180/s1, Figure S1: KEGG enrichment analysis of the first cluster of genes from the time-course analysis; Figure S2: KEGG enrichment analysis of the second cluster of genes from the time-course analysis; Table S1: Primers used for qRT-PCR in this study.
