# Supplementary material for: Comparative Transcriptomic Analysis Provides Insight into Spatiotemporal Expression Patterns of Pivotal Genes During Critical Growth Stages in Min Pig Breed
Source: Biomolecules. 2025 Jan 26;15(2):180. doi: 10.3390/biom15020180 (PMC11853420; doi:10.3390/biom15020180)
Supplement: Supplementary file 1 [file biomolecules-15-00180-s001.zip › biomolecules-supplementary figure.pdf]

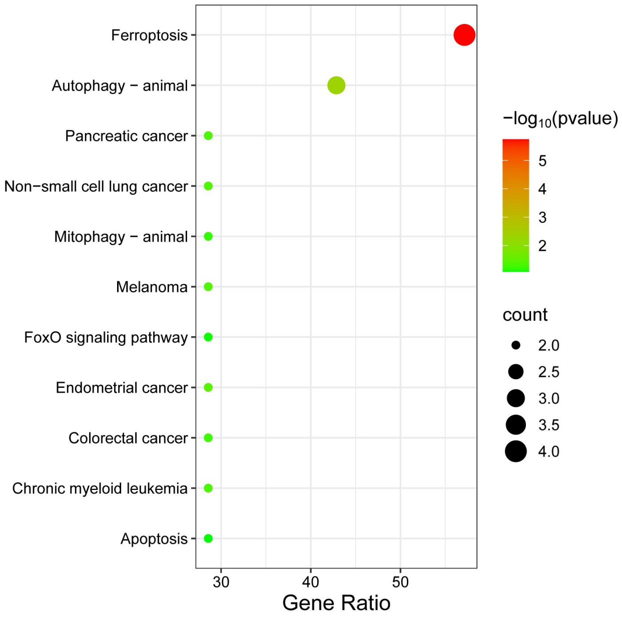

Supplementary Figures S1. KEGG enrichment analysis of the first cluster of genes from the time-course analysis.

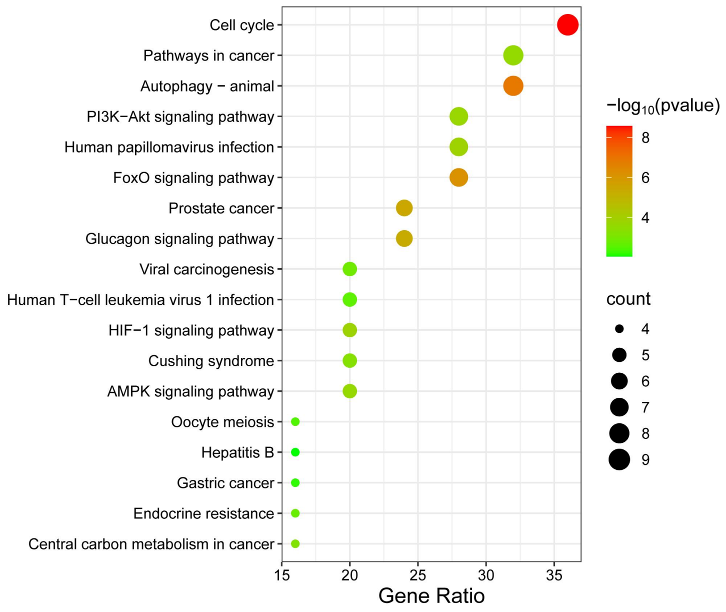

Supplementary Figures S2. KEGG enrichment analysis of the second cluster of genes from the time-course analysis. (displaying pathways with $P < 0.01$)
